# Intranasal Dexmedetomidine as a Sedative Premedication for Patients Undergoing Suspension Laryngoscopy: A Randomized Double-Blind Study

**DOI:** 10.1371/journal.pone.0154192

**Published:** 2016-05-19

**Authors:** Chengxiang Lu, Li-Ming Zhang, Yuehong Zhang, Yanlu Ying, Ling Li, Lixin Xu, Xiangcai Ruan

**Affiliations:** 1 Department of Anesthesiology, First Municipal People’s Hospital of Guangzhou, an Affiliate Hospital of Guangzhou Medical College, Guangzhou, China; 2 University of Pittsburgh Medical Centre, Pittsburgh, Pennsylvania, United States of America; 3 Department of Ophthalmology, First Municipal People’s Hospital of Guangzhou, an Affiliate Hospital of Guangzhou Medical College, Guangzhou, China; 4 Department of Medical Infomation, First Municipal People’s Hospital of Guangzhou, an Affiliate Hospital of Guangzhou Medical College, Guangzhou, China; Center for Rheumatic Diseases, INDIA

## Abstract

**Background:**

Intranasal dexmedetomidine, a well-tolerated and convenient treatment option, has been shown to induce a favorable perioperative anxiolysis in children. We investigate intranasal dexmedetomidine as a sedative premedication for anesthesia recovery in an adult population.

**Methods:**

A prospective randomized controlled trial; 81 adult patients scheduled for elective suspension laryngoscopy received intranasal dexmedetomidine (1 μg∙kg^–1^) or a placebo 45–60 min before anesthetic induction. Extubation time was used as the primary outcome measure. Secondary variables included the levels of sedation (Observer’s Assessment of Alertness/Sedation scale, OAA/S) and anxiety (4-point anxiety score), anesthetic and analgesic requirements, hemodynamic fluctuations, and anesthesia recovery as well as side effects.

**Results:**

The levels of sedation and anxiety differed significantly between the two groups at anesthesia pre-induction (*p* < 0.001 and = 0.001, respectively). Repeated-measure general linear model determined no significant interaction effect between group and time on the targeted concentration of propofol (F = 1.635, *p* = 0.200), but a significant main effect of group existed (F = 6.880, *p* = 0.010). A moderate but significant decrease in the heart rate was recorded in the dexmedetomidine group at pre-induction. Episodes of tachycardia and hypertension after tracheal intubation and extubation were more frequent in the placebo group.

**Conclusions:**

Intranasal dexmedetomidine as a sedative premedication induced a favorable perioperative anxiolysis without prolongation in anesthesia recovery; the hemodynamic effect was modest.

**Trial Registration:**

ClinicalTrials.gov NCT 02108171

## Introduction

Dexmedetomidine (DEX) is a highly selective, short-acting, alpha 2-adrenoreceptor agonist. It can provide sedative, analgesic, and anxiolytic effects with minimal respiratory depression, which makes it an almost-perfect adjuvant for anesthesia, as well as an ideal candidate for relieving anxiety or nervousness before anesthesia. However, reports of adverse hemodynamic complications, including several cases of cardiac arrest, might have hindered the widespread use of DEX [1,2].

It has been suggested that a smaller dose or routes other than rapid intravenous delivery may help minimize the hemodynamic risk of DEX [3,4,5,6]. The intranasal route is a convenient and effective method of administration for many medications, and intranasal DEX has been shown to have a high rate of patient acceptance [7,8,9,10]. Recently, several pediatric studies reported beneficial perioperative outcomes of premedication with intranasal DEX [11,12,13,14,15,16], which may indicate that it could be an alternative to traditional premedication for the pediatric population.

For adult patients, physicians might be reluctant to prescribe sedative premedications due to concerns of delayed recovery from anesthesia. However, a Cochrane review found that there is no evidence to support such concerns in the ambulatory setting [17]. The proper use of premedication may actually decrease anesthetic and analgesic requirements as well as some side effects, such as postoperative emesis, and thereby ultimately provide benefits in selected surgical patients [18]. There is no known link between delayed recovery and intraoperative DEX [19,20,21]. There are, however, no scientific studies that have specifically investigated a link between using DEX as a sedative premedication and delayed recovery from anesthesia. One recent study compared intranasal DEX with either intranasal ketamine or a placebo in children undergoing procedural sedation and found that both intranasal premedications were more sedative and decreased times to wake and discharge compared to the placebo [22].

We conducted a randomized, double-blind, placebo-controlled study in patients who underwent suspension laryngoscopic surgery to examine the effects of 1 μg∙kg^–1^ of intranasal DEX on their anesthesia recovery. Previous studies conducted on healthy volunteers have indicated that the studied intranasal dose is well tolerated [10,23]. Our primary outcome measurement was the recovery time from general anesthesia, and other outcomes included the levels of sedation and anxiety, anesthetic and analgesic requirements, hemodynamic parameters, postoperative side effects, and patient satisfaction.

## Materials and Methods

Following approval by the Ethics Committee of Guangzhou First Municipal People’s Hospital, the study protocol was registered at clinicaltrials.gov (registration number NCT02108171). The study protocol and supporting CONSORT checklist are available as Supporting Information; see S1–S3 Files.

American Society of Anesthesiologists (ASA) patients with a physical status of I or II, body mass index (BMI)<30 kg/m^2^, age between 18 and 60 years, scheduled for elective suspension laryngoscopy for the removal of benign lesions under general anesthesia between March and June 2014 were screened for eligibility at the pre-anesthesia consultation. Written informed consent was obtained from all patients at least one day before surgery. Exclusion criteria included the following: 1) a known allergy or hypersensitivity to DEX or other anesthetics; 2) a previous history of heart disease; 3) a heart rate (HR) <45 beats per minute (bpm); 4) a second- or third-degree atrioventricular block; 5) pregnant or breast-feeding women; 6) premenopausal women without reliable contraception; 7) patients on antihypertensive drugs, such as α-methyldopa, clonidine, or other α_2_-adrenergic agonists; 8) asthma, 9) sleep apnea syndrome; 10) organ dysfunction; 11) patients with mental illness; and 12) the long-term use of sedatives and analgesics.

A computer-generated list of numbers accomplished by a research member (L.L.) was used for drug allocation. The list was concealed in opaque sealed envelopes that were numbered and opened sequentially after obtaining the patients’ consent. A nurse who was not involved in any other part of the study obtained the envelopes and then prepared the premedications by drawing 1 μg∙kg^–1^ of DEX (Hengrui Med, Jiangsu, China; original concentration 100 μg∙ml^–1^) into a 1-mL syringe and diluting it to a volume of 1 mL with 0.9% saline or the same volume of saline as a control (CON). The appearance of DEX and CON premedications cannot be differentiated by a third person. Test premedications (DEX or CON) were dripped into both nostrils of the patients in a supine head down position about 45 to 60 minutes before anesthesia induction. The patients fasted for at least 6 h and were not given other premedications. They were taught to rate their anxiety levels using a 4-point anxiety score (1 = combative, 2 = anxious, 3 = calm, and 4 = amiable) and satisfaction using a 3-point satisfaction score (1 = highly satisfactory, 2 = acceptable, and 3 = unacceptable). A blinded observer assessed sedation with a 7-point modified Observer’s Assessment of Alertness/Sedation scale (OAA/S, appendix). HR and oxygen saturation (SpO_2_) were monitored by fiber-optic pulse oximetry during patient transfer from the ward to the operating room.

When patients arrived in the operating room, ASA standard monitoring and Narcotrend^®^ electroencephalography (software version 4.3; Monitor Technik, Bad Bramstedt, Germany) were placed and recorded. Approximately 40 min following the administration of the test medications, sedation and anxiety levels were recorded. Subsequently, total intravenous anesthesia was induced via the target-controlled infusion (TCI) of propofol and remifentanil with a supply of oxygen. After the loss of consciousness, patients received 0.6 mg∙kg^–1^ of rocuronium followed by mask ventilation; the endotracheal tube was placed 1 min later. Mechanical ventilation was adjusted to an end-tidal carbon dioxide concentration (P_ET_CO_2_) of 4.0–4.7 kPa.

Propofol was infused intraoperatively to a TCI plasma concentration of 2.5 μg∙ml^–1^ using Diprifusor^TM^ software (version 2.0, Graseby^®^ 3500 anesthesia pump; Smiths Medical, Watford, UK). The infusion rate was adjusted via plasma concentration increments of 0.5 μg∙ml^–1^ at 2-min intervals to maintain the Narcotrend index between ‘D0’ and ‘E1’ until the end of surgery. Remifentanil was infused to achieve a TCI plasma concentration of 3.0 ng∙ml^–1^ using the Minto pharmacokinetic model [24] and then adjusted to maintain the systolic blood pressure (SBP) at 25% of the pre-operative value and the HR at less than 90 bpm. To maintain a neuromuscular blockade, 0.15 mg∙kg^–1^ increments of rocuronium were infused upon observation of the first twitch in a train-of-four response with the nerve stimulator. When the operative laryngoscope was removed, the propofol and remifentanil infusions were stopped simultaneously.

After surgery, the patients were transferred to the post-anesthesia care unit (PACU) and administered neostigmine and atropine to reverse the residual neuromuscular blockade according to the nerve stimulator. Assisted ventilation was stopped when adequate ventilation was confirmed: a respiratory rate (RR) >8 bpm and SpO_2_ >90% on air for more than 5 min. The endotracheal tube was extubated when patients met the extubation criteria and could follow commands by squeezing the anesthetist’s hand.

Baseline measurements of sedation and anxiety status, non-invasive blood pressure, HR, and oxygen staturation were collected immediately before and repeatedly after intranasal DEX or placebo administration. The durations between intranasal dexemedetomidine and anesthesia intubation as well as between anesthesia intubation and the cessation of anesthetics infusion were recorded. In addition, we recorded the target concentrations of propofol and remifentanil at induction (T1), before insertion (T2), and upon removal of the operative laryngoscope (T3), at the return of spontaneous breathing (T4), at emergence (T5), and at extubation (T6). The time elapsed between the cessation of anesthetic infusions and extubation was recorded as the recovery time. Patient satisfaction scores were collected when patients were discharged from the PACU. Adverse events in the peri-operative period included hypertension (SBP increased 130% of the pre-operative value for more than 1 min), hypotension (SBP decreased more than 30% of the pre-operative value for more than 1 min), tachycardia (HR >100 bpm for more than 10 s), bradycardia (HR <45 bpm for more than 10 s), shivering, nausea, or vomiting. When clinically indicated, 5 mg of ephedrine or 0.5 mg of atropine was administered intravenously. Respiratory depression (RR <8 bpm and SpO_2_ <90%) and analgesic requests within 2 h after extubation were recorded. In addition, an investigator who was blinded from the grouping (Y.Y.) asked the patients to mark their pain level on a 0–100 mm visual analogue scale (VAS). A VAS above 50 mm was treated with intravenous 40 mg of parecoxib. Nausea or vomiting was treated with 4 mg of intravenous ondansetron.

Power analysis for two-group independent Student’s t-test was performed based on the results of our pilot study in which the mean (standard deviation, SD) time to extubation was 18 (5) min. To detect a 20% difference in the time to extubation between the control and treatment groups with a 5% type I error and a power of 0.9 at a 2–sided *P* value < 0.05, a minimum sample size of 39 patients per group would be required. We recruited 81 patients, considering a 4% dropout rate

Statistical analyses were performed using SPSS 17.0 (SPSS Inc., Dover, Delaware, USA). Descriptive statistics such as mean ± SD, median (extreme range), and proportions were used to analyze patient characteristics. Assumptions of normal distribution were tested for all of the continuous variables via Shapiro-Wilk test. Student’s two-sample *t* test was used to analyze the anesthesia recovery times. Mann–Whitney *U* test was used to compare the time durations between drug administration and anesthesia intubation, anesthesia, surgery, and the OAA/S sedation score. Repeated-measure general linear model (GLM) was calculated to assess differences in the target concentrations of remifentanil and propofol between treatments over time. When there was an interaction between group and time on the dependent variable, a pairwise comparisons with Bonferroni adjustment was performed for each time point. The numbers of patients rating anxiety score > 2 and satisfaction score < 2, and the episodes of adverse events, were analyzed using Fisher’s exact tests. A two-tailed *p* value less than 0.05 was considered statistically significant.

## Results

**Fig 1:**
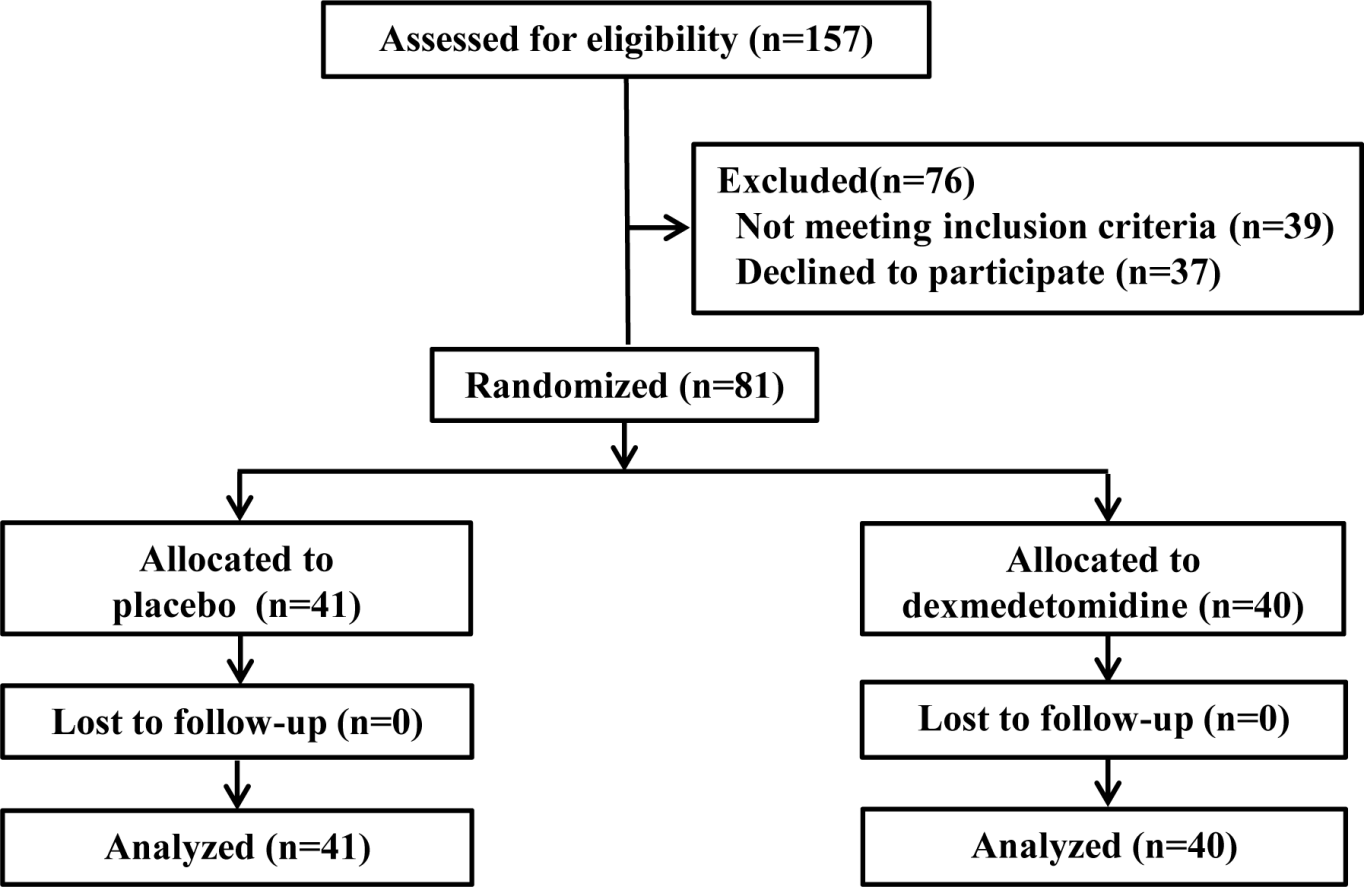
Patient-flow diagram.

We initially assessed 157 patients for eligibility. After excluding the patients who did not meet the inclusion criteria or declined to participate, we recruited 81 patients for this randomized study, with 41 patients allocated in the CON group and 40 patients in the DEX group, and all the allocated patients completed this study (Fig 1). Patients’ baseline characteristics and surgical data were similar between the groups (Table 1).

**Table 1:** Baseline characteristics and surgical data of patients receiving intranasal placebo or dexmedetomidine. Values are in mean (standard deviation), number (percentage), or in median (extreme range) where appropriate.

	Placebo	Dexmedetomidine
	(n = 41)	(n = 40)
Age (yr)	43.6 (9.2)	45.9 (10.1)
Weight (kg)	60.1 (10.4)	59.4 (9.1)
Height (cm)	162.2 (6.1)	162.5 (8.0)
Sex (Male: Female)	13 (31.7%): 28 (68.3%)	16 (40.0%): 24 (60.0%)
ASA status (I: II)	38 (92.7%): 3 (7.3%)	36 (90.0%): 4 (10.0%)
Duration from intranasal drug administration to arrival at operating room (min)	40 [0–78]	40 [5–100]
Duration from intranasal drug administration to anesthesia intubation (min)	54 [23–115]	55 [32–141]
Duration of anesthesia	38 [18–158]	35[16–87]
Duration of surgery (min)	18 [5–132]	18 [4–61]

As shown in Table 2, the modified OAA/S scores were comparable among groups at baseline and reached significantly different at the pre-induction of anesthesia (*p* < 0.001); after extubation, there was no difference in sedation. Compared to the placebo group, the dexmedetomidine group had more patients rated anxiety score >2 at the pre-induction (*p* = 0.001), and scored highly satisfactory (*p* = 0.015).

**Table 2:** Modified OAA/S, anxiety, and satisfaction scores of patients receiving intranasal placebo or dexmedetomidine. Values show median (interquartile range) or number of patients (percentage).

	Placebo	Dexmedetomidine	*P* Value
	(n = 41)	(n = 40)	
**Modified OAA/S score**			
Before intranasal drugs	6 [6–6]	6 [6–6]	0.317
Pre-induction	6 [5–6]	4 [4–5]	< 0.001
After extubation	4 [3–5]	4 [3–4]	0.776
**Anxiety score > 2**			
Before intranasal drugs	23 (56.1%)	25 (62.5%)	0.558
Pre-induction	29 (70.7%)	39 (97.5%)	0.001
**Satisfaction score < 2**	25 (61.0%)	34 (85.0%)	0.015

**Fig 2:**
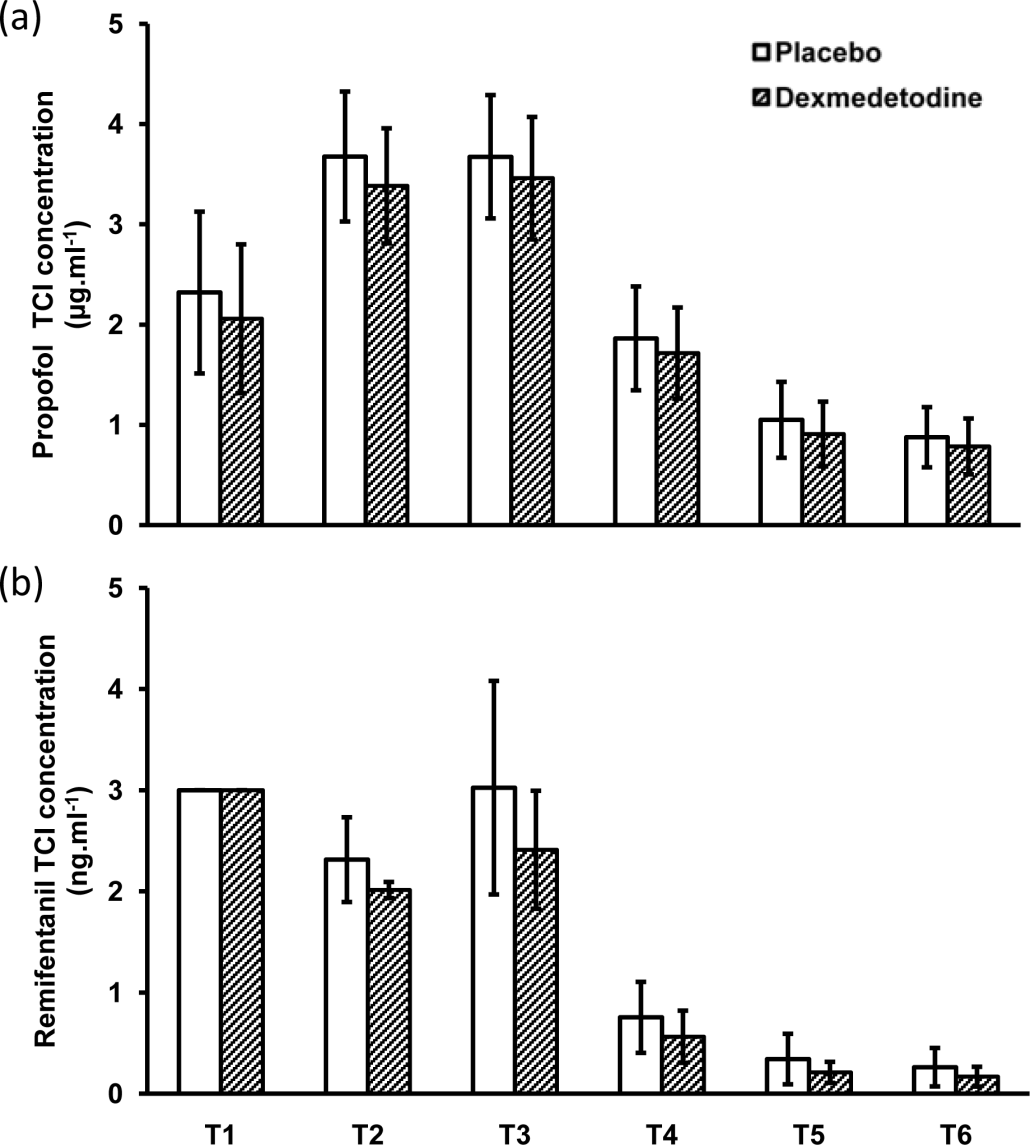
Predicted effect-site concentrations of propofol (a) and remifentanil (b) after intranasal placebo or dexmedetomidine.The target concentrations of propofol or remifentanil were recorded at induction (T1), before insertion (T2), and upon removal of the operative laryngoscope (T3), at the return of spontaneous breathing (T4), at emergence (T5), and at extubation (T6). TCI stands for target-controlled infusion. Error bars represent standard deviation.

By using the repeated-measure GLM, we found a significant interaction effect between group and time on the targeted concentration of remifentanil (F = 9.255, *p* = 0.001), and a significant main effect of group (F = 21.402, *p* < 0.001). The pairwise comparisons of the concentration of remifentanil showed that each time point significantly differed from each other time point (all *p* < 0.001). There was no significant interaction effect between group and time on the targeted concentrations of propofol (F = 1.635, *p* = 0.200), but the main effect of group was significant (F = 6.880, *p* = 0.010), indicating intranasal dexmedetomidine reduced anesthetics requirement of propofol. (Fig 2).

**Fig 3:**
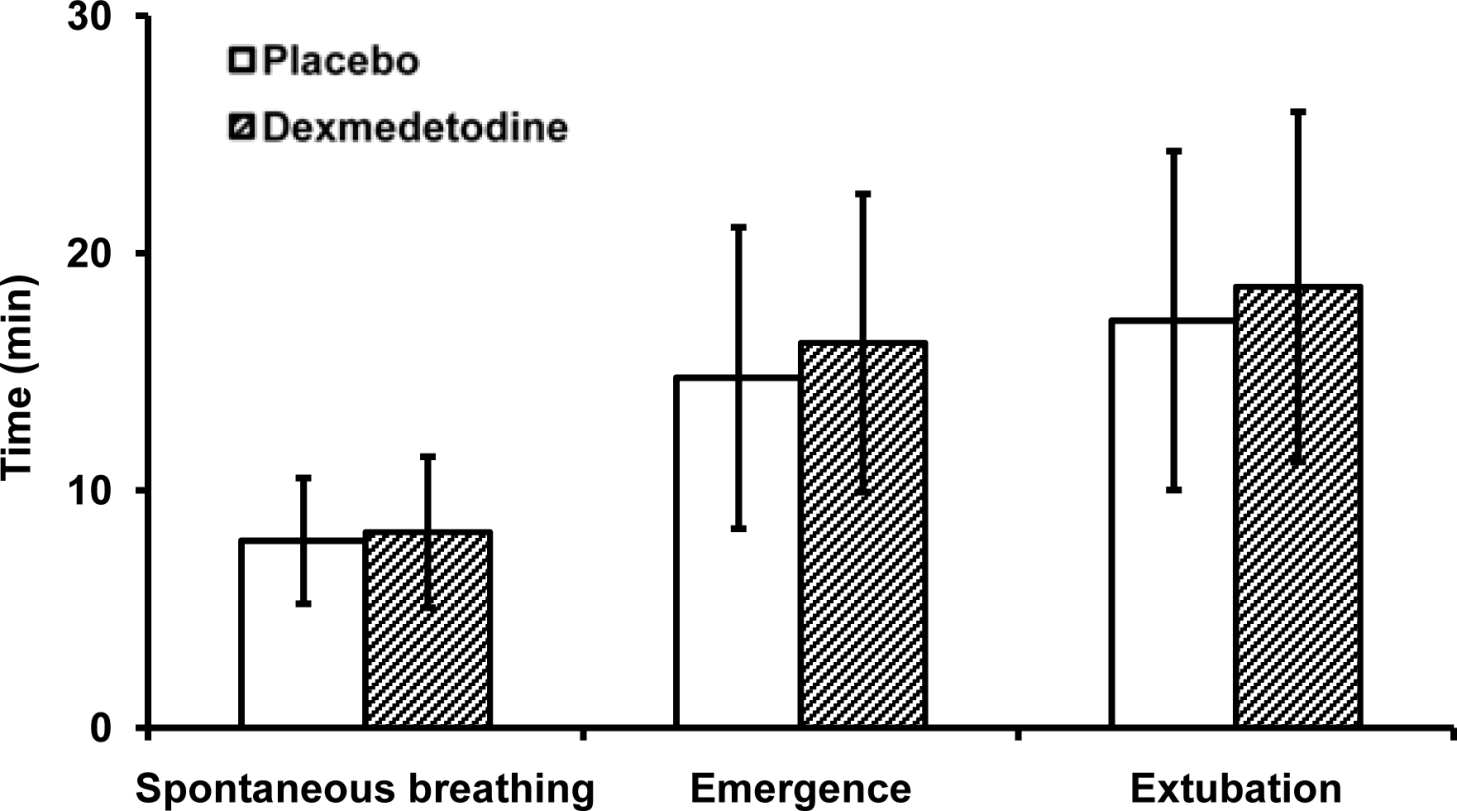
Anesthesia recovery times were similar between patients receiving intranasal placebo and those receiving dexmedetomidine. The times elapsed between stopping anesthetic infusions and adequate ventilation, consciousness, and extubation were also recorded as time to spontaneous breathing, emergency, and time to extubation. Error bars represent standard deviation.

There were no significant differences noted in the time period of returning to spontaneous breathing, conscious emergence, and tracheal extubation between the groups (Fig 3).

No significant difference in mean arterial pressure (MAP) was found between the groups. A significant decrease in HR was noted in the DEX group at pre-induction compared to the CON group, but no decrease of more than 20% of the baseline value, which was required for clinical intervention, was recorded in either group at any time (S1 Fig). There were no significant differences between the groups with regard to episodes of bradycardia and hypotension. However, in the placebo patients, episodes of tachycardia and hypertension increased significantly after tracheal intubation and extubation compared to the DEX group (Table 3). Fifteen patients in the CON group and five patients in the DEX group required postoperative analgesia (*p* = 0.005). Three patients from the CON group and one patient after intranasal DEX experienced nausea (*p* = 0.623), but only one patient in the CON group was recorded as vomiting (*p* = 1.0). Four patients in the CON group and one in the DEX group reported the occurrence of postoperative shivering (*p* = 0.371). No intraoperative awareness was recalled.

**Table 3:** Patients with cardiovascular adverse episodes in two groups. Values represent numbers of patients.

	Placebo	Dexmedetomidine	*P* Value
	(n = 41)	(n = 40)	
**Bradycardiac episode**			
Pre-induction	0	2	0.241
After intubation	1	1	1
Intraoperative	5	3	0.737
After extubation	5	7	0.502
**Tachycardiac episode**			
Pre-induction	0	0	1
After intubation	8	1	0.037
Intraoperative	11	5	0.105
After extubation	9	2	0.026
**Hypotensive episode**			
Pre-induction	0	2	0.241
After intubation	3	4	0.973
Post-induction	3	4	0.973
After extubation	1	0	1
**Hypertensive episode**			
Pre-induction	1	0	1
After intubation	6	0	0.037
Intraoperative	6	5	0.779
After extubation	14	5	0.022

## Discussion

The principal finding of this study was that a 1-μg∙kg^–1^ intranasal dose of DEX premedication did not delay anesthesia recovery in laryngoscopic patients. In addition, we found a number of benefits of intranasal DEX premedication, including perioperative anxiolysis, less anesthetic requirements, stable hemodynamics, and improved patient satisfactory outcomes.

Similar to the present study, other research on premedication with ɑ2 agonists have indicated no delayed recovery in the outcome. These investigations have included pre-anesthetic oral clonidine among children [25] and adult patients undergoing coronary bypass [26] or general surgery [27] as well as using 0.5–1.0 μg∙kg^–1^ of intravenous DEX [4,28,29]. However, mixed results in recovery from premedication with both oral clonidine [30] and intravenous DEX [31] have been reported. Intranasal administration, a noninvasive and highly convenient method, has been used successfully to deliver DEX to children. At an appropriate dose, intranasal DEX premedication has been associated with similar effects on anesthesia recovery time as oral midazolam [12,13] or a placebo [16] in children. Previous studies have reported that intranasal DEX 1–1.5μg∙kg^–1^ produced significant sedation within 45–60 min and its elimination half-life was 114 min in healthy volunteers[7,8,9,10]. The current study was the first randomized controlled study to determine the effects of intranasal DEX premedication on adult patients’ recovery from general anesthesia.

Several mechanisms may explain a few of the adverse effects of DEX premedication on anesthesia recovery in the present study. First, the anesthetic-sparing properties and analgesic qualities of DEX have been positively associated with anesthesia recovery. Its additive or synergistic role in intraoperative anesthesia and its potential analgesic effect of alleviating postoperative pain may expedite early recovery. Regarding residual sedation, intranasal DEX premedication may have the same residual effect in the immediate postoperative period as its intravenous administration. Previous data from healthy volunteers revealed that both administration routes of DEX had a similar elimination half-life (t_1/2_) with a median (range) of 114 (107–151) and 115 (99–145) min, respectively, and had comparable sedation effects as well [10]. A multicenter trial assessed DEX by intravenous loading doses and maintenance infusion for procedure sedation and reported a favorable recovery outcome over placebo [32]. It is likely that a pre-anesthetic intranasal administration would leave a lower accumulated plasma concentration of DEX or less residual sedation in the immediate postoperative period than an intravenous loading dose followed by a maintenance infusion. Furthermore, the use of a small dose of DEX intranasally may also contribute to a favorable recovery. We and others have previously reported this favorable association between low-dose DEX given intravenously at pre-anesthetic time and anesthesia recovery [4,28,29]. In the present study, intranasally low-dose DEX administration, as previously described elsewhere [7,8,9], induced mild but clinically favorable sedation compared to the placebo. Although a larger dose of intranasal DEX has been shown to be more effective in pre-anesthetic sedation, the expense is that it would delay recovery in pediatric patients [16].

The hemodynamic parameters of intranasal DEX are noteworthy. Reports of hypotension, bradycardia, and significant cardiovascular dysfunction have been associated with DEX infusion. In this study, we also noted a decrease in HR and SBP at anesthesia pre-induction in the DEX-premedicated patients. However, the decrease in both HR and SBP was mild or moderate and well tolerated in our relatively young and healthy patients without requirement of clinical intervention. Previous studies using intranasal DEX reported similar manageable hemodynamic responses [7,8,9,10]. In addition, we found that the hemodynamic response to tracheal intubation and extubation was attenuated in the DEX-premedicated patients. We speculate that the dose reduction and the specified intranasal route of administration may help mitigate the hemodynamic instabilities of DEX. A case report published at the completion of this clinical trial [33] noted that a high dose of intranasal DEX (~2.4 μg∙kg^–1^) caused severe bradycardia in a pediatric patient, indicating that a large intranasal dose of DEX to achieve deep sedation should be administrated cautiously.

This study has several limitations. First, sprayed or atomized delivery can markedly improve uptake, the time to onset, and the pharmacological efficacy of intranasal medication when compared with the syringe nasal drops used in our study. Nevertheless, the syringe nasal method has been used in many studies and has been found to be a cost-effective drug delivery technique in experienced hands and with cooperative patients [34,35]. Second, this study involved relatively healthy and young participants. Although there have been a few reports on the pharmacokinetics and pharmacodynamics of intranasal DEX, the absolute bioavailability remains as yet largely under-investigated. The effects of intranasal DEX may differ with co-existing diseases, elderly patients, and various surgical stimulations. Third, target concentrations were used to assess the intraoperative requirements of propofol and remifentanil. If the plasma concentrations were to be measured, the anesthetic and analgesic sparing effects would be more convincing. However, the pharmacokinetic models we applied are now well established. Four, Although none of the enrolled patients had epistaxis, ulcer, infection, deviated septum, or trauma, criteria should exclude those patients with any of these oblivious nasal abnormality before trial recruitment.

In conclusion, intranasal DEX at a dose of 1 μg∙kg^–1^ as a sedative premedication has demonstrated a favorable perioperative anxiolysis without prolongation in anesthesia recovery, and its hemodynamic effect was well tolerated by the patients included in this study. Intranasal DEX can be considered another premedication alternative to manage selected patients with pre-anesthesia anxiety.

## Appendix

Modified Observer’s Assessment of Alertness/Sedation Scale

6 Appears alert and awake, responds readily to name spoken in normal tone
5 Appears asleep but responds readily to name spoken in normal tone
4 Lethargic response to name spoken in normal tone
3 Responds only after name is called loudly or repeatedly
2 Responds only after mild prodding or shaking
1 Does not respond to mild prodding or shaking
0 Does not respond to noxious stimulus

## Supporting Information

S1 Fig Haemodynamics fluctuations. Heart rate (a) and mean arterial pressure fluctuations (b) after intranasal placebo or dexmedetomidine. In comparison with baseline levels, heart rate increased at T4–5 in the placebo group (p = 0.000 and 0.023, respectively) but decreased at T2–3 and T7–10 in the dexmedetomidine group (*p* = 0.038, 0.002, 0.011, 0.034, 0.001, and 0.003, respectively). No significant difference in mean arterial pressure between the groups. In comparison with baseline levels, mean arterial pressure increased at T2, 7–10 in the placebo group (*p* = 0.004, 0.033, 0.000, 0.000, 0.003, respectively) and at T9 in the dexmedetomidine group (*p* = 0.019). Data points were shifted horizontally to avoid overlapping. Error bars represent standard deviation. T1, before intranasal drops; T2, on arrival at the operating room; T3, at pre-induction; T4, after tracheal intubation; T5, after inserting operative laryngoscope; T6, after removal of laryngoscope; T7, on arrival at the post-anaesthesia unit; T8, at emergency; T9, after extubation; T10, before leaving the post-anaesthesia unit.
(DOCX)

S1 File Consort Checklist. (DOCX)

S2 File Protocol translation. This is trial protocol in English.
(DOCX)

S3 File Protocol. This is original protocol in Chinese
(DOCX)

S4 File Informed Consent Form. This is original Informed Consent Form for this trial.
(DOCX)
