# Supplementary material for: Intranasal Dexmedetomidine as a Sedative Premedication for Patients Undergoing Suspension Laryngoscopy: A Randomized Double-Blind Study
Source: PLoS One. 2016 May 19;11(5):e0154192. doi: 10.1371/journal.pone.0154192 (PMC4873234; doi:10.1371/journal.pone.0154192)

**Supplementary material**

**FIGURE LEGEND**

Heart rate(a) and mean arterial pressure(b) fluctuations after intranasal placebo(
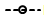
) or dexmedetomidine(
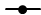
).In comparison with baseline levels, heart rate increased at T4–5 in the placebo group (p = 0.000 and 0.023, respectively) but decreased at T2–3 and T7–10 in the dexmedetomidine group (*p* = 0.038, 0.002, 0.011, 0.034, 0.001, and 0.003, respectively). No significant difference in mean arterial pressure between the groups. In comparison with baseline levels, mean arterial pressure increased at T2, 7–10 in the placebo group (*p* = 0.004, 0.033, 0.000, 0.000, 0.003, respectively) and at T9 in the dexmedetomidine group (*p* = 0.019). Data points were shifted horizontally to avoid overlapping. Error bars represent standard deviation. T1, before intranasal drops; T2, on arrival at the operating room; T3, at pre-induction; T4, after tracheal intubation; T5, after inserting operative laryngoscope; T6, after removal of laryngoscope; T7, on arrival at the post-anaesthesia unit; T8, at emergency; T9, after extubation; T10, before leaving the post-anaesthesia unit.

**FIGURE S1**


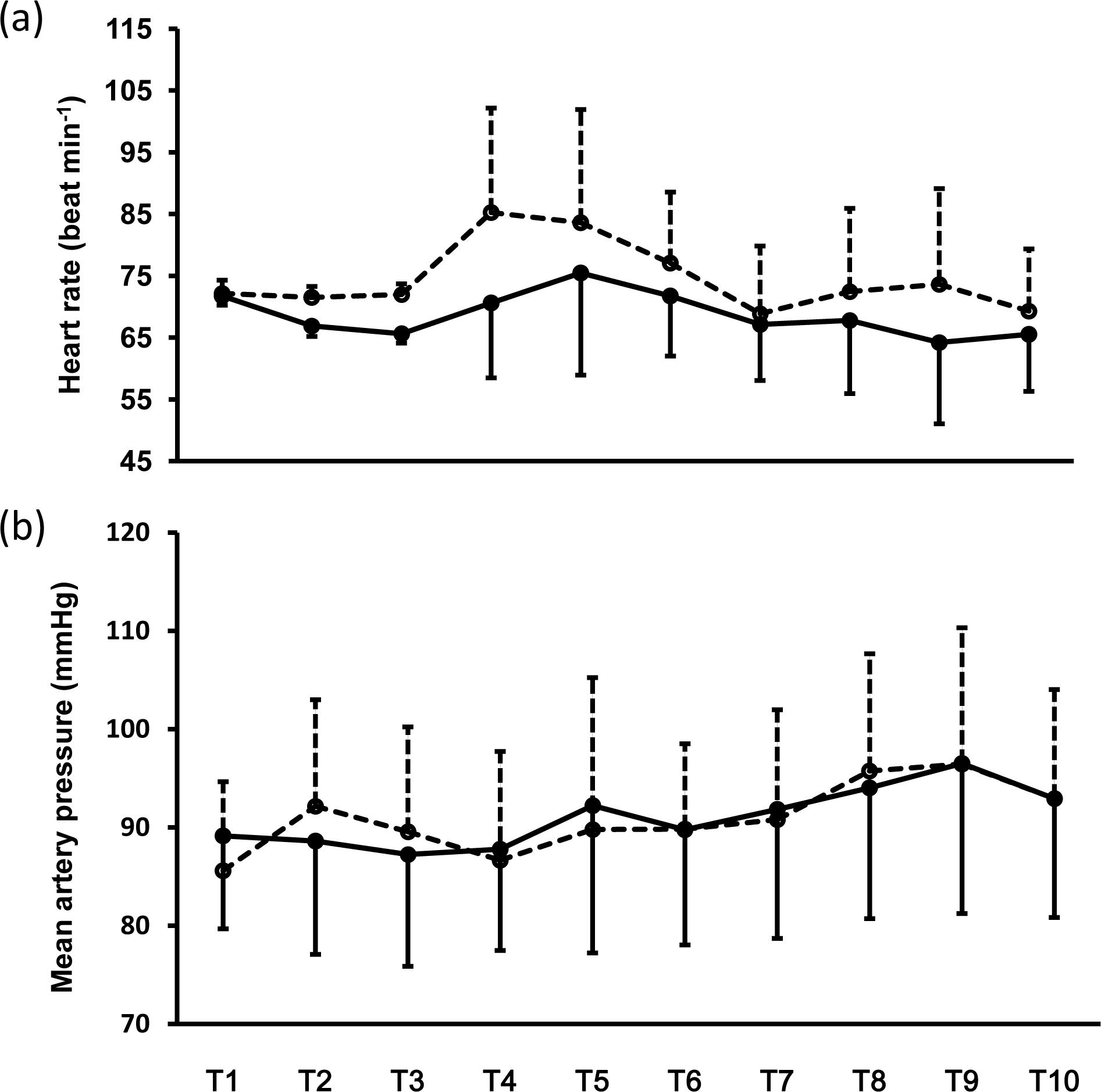

Supplement: S1 Fig — Heart rate (a) and mean arterial pressure fluctuations (b) after intranasal placebo or dexmedetomidine. In comparison with baseline levels, heart rate increased at T4–5 in the placebo group (p = 0.000 and 0.023, respectively) but decreased at T2–3 and T7–10 in the dexmedetomidine group (p = 0.038, 0.002, 0.011, 0.034, 0.001, and 0.003, respectively). No significant difference in mean arterial pressure between the groups. In comparison with baseline levels, mean arterial pressure increased at T2, 7–10 in the placebo group (p = 0.004, 0.033, 0.000, 0.000, 0.003, respectively) and at T9 in the dexmedetomidine group (p = 0.019). Data points were shifted horizontally to avoid overlapping. Error bars represent standard deviation. T1, before intranasal drops; T2, on arrival at the operating room; T3, at pre-induction; T4, after tracheal intubation; T5, after inserting operative laryngoscope; T6, after removal of laryngoscope; T7, on arrival at the post-anaesthesia unit; T8, at emergency; T9, after extubation; T10, before leaving the post-anaesthesia unit. (DOCX) [file pone.0154192.s001.docx]
