# Supplementary material for: Intranasal Dexmedetomidine as a Sedative Premedication for Patients Undergoing Suspension Laryngoscopy: A Randomized Double-Blind Study
Source: PLoS One. 2016 May 19;11(5):e0154192. doi: 10.1371/journal.pone.0154192 (PMC4873234; doi:10.1371/journal.pone.0154192)
Supplement: S2 File — This is trial protocol in English. (DOCX) [file pone.0154192.s003.docx]

**Protocol_S1 translation**

**A randomised controlled trial of intranasal dexmedetomidine as premedication for suspension laryngoscopy**

**Background**

Most of the patients have preoperative varying degrees of stress and anxiety, which enhance the stress response of the patients and affect the normal conduct of anesthesia and surgery. In addition, hyperexcitability of the sympathetic system would cause adverse cardiovascular events, which affect the postoperative recovery. Therefore, it is necessary to use some anti-anxiety and sedative drugs pre-operation. Premedication, having the advantages of sedative, anxiolytic and less adverse reactions, is an important component of anesthesia and the beginning of anesthesia^[1]^. As the traditional premedication, Phenobarbital has some shortcomings, such as less sedative and weak anxiolytic. Having superior sedative, hypnotic and anterograde amnesia than phenobarbital, Midazolam is the first choice for premedication in most developed countries^[2~3]^. However, Midazolam is accompanied by inhibition of respiration, excessive sedation, easily induced delirium, wake prolonged recovery and other shortcomings^[5~6]^, which exist certain security risks and may result in delayed recovery in the absence of monitoring conditions. That is why the application of Midazolam is limited.

Dexmedetomidine (DEX) is a highly selective, short-acting, alpha 2-adrenoreceptor agonist. It can provide s sedative, analgesic, and anxiolytic effects, inhibiting the sympathetic, hemodynamic stability and save narcotic drugs with minimal respiratory depression, which makes it an almost perfect adjuvant for anesthesia, as well as an ideal candidate for relieving anxiety or nervousness before anaesthesia. During anesthesia recovery, DEX can reduce the incidence of postoperative nausea, vomiting, shivering, and agitation, also can reducing the need for analgesic drugs and improve the quality of anesthesia recovery as well as patient satisfaction^[7~10]^.

However, reports of adverse haemodynamic complication, including several cases of cardiac arrest, might have hindered the widespread use of DEX. As premedication, DEX has a variety of modes of administration, such as, intramuscular injection, intranasal administration. Intravenous injection of dexmedetomidine would impact on circulatory system. In addition, continuous infusion may result in delayed recovery^[11~12]^. Intravenous injection and intramuscular injection are invasive, which contrary to noninvasive premedication. Dexmedetomidine is colorless, odorless, non-mucosal stimulation, convenient intranasal administration, good patient acceptance and comfort. Recently, several paediatric studies reported beneficial perioperative outcomes of premedication with intranasal DEX. Yuen VM, et al found that approximately 75% and 92% of subjects attained clinically significant sedation after 1 and 1.5μg∙kg^–1^ of intranasal dexmedetomidine respectively^[13]^. N. Nooh, et al show that intranasal DEX for sedation during unilateral third molar surgery under local anaesthesia is effective and safety^[14]^. Recently, several paediatric studies reported beneficial perioperative outcomes of premedication with intranasal DEX^[15]^. Therefore, intranasal DEX may indicate that it could be an alternative to traditional premedication.

For adult patients, physicians might be reluctant to prescribe sedative premedications due to concerns of delayed recovery from anaesthesia. However, a Cochrane review found that there is no evidence to support such concerns in the ambulatory setting^[16]^. Proper use of premedication may actually decrease anaesthetic and analgesic requirements as well as some side effects, such as postoperative emesis, and thereby ultimately provide benefits in selected surgical patients[^17]^. There is no known link between delayed recovery and intraoperative DEX. There are, however, no scientific studies that have specifically investigated a link between using DEX as a sedative premedication and delayed recovery from anaesthesia.

The purpose of the study is to study sedative effect, safety and the impact of anesthesia recovery period of intranasal dexmedetomidine premedication for suspension laryngoscopy

**Study Design**

The study is a randomised, double-blind, placebo-controlled study in patients. Following approval by our hospital institutional ethics committee, the study protocol will be registered at clinicaltrials.gov. All patients taking part in the study must provide their written informed consentt least one day before surgery.

**Study Type:**  Interventional

**Allocation:** Randomization

**Endpoint classification:** Safety and efficacy study

**Intervention:** Parallel assignment

**Blinding:**  Double-blind

**Eligibility**

**Age eligible for study:** 18 years to 60 years

**Genders eligible for study:** Both

**Receive health volunteers:** No

**Criteria**

**Inclusion criteria**

Surgery: The laryngoscope vocal polyp excision

Aged between 18 to 60 years old

Body mass index（BMI）<30kg/m^2^

American Society of Anesthesiologist (ASA) I - II.

**Exclusion criteria**

The investigator refused to participate

Known allergy or hypersensitivity reaction to DEX or other anaesthetics;

Previous history of heart disease;

Pregnant women;

Premenopausal women without reliable contraception

Preoperative heart rate (HR) <45 beats per minute (bpm);

Second- or third-degree atrioventricular block;

Ischemic heart disease;

Taking antihypertensive drugs, such as α-methyldopa, clonidine, or other α_2_-adrenergic agonists;

Asthma;

Sleep apnoea syndrome;

Liver and kidney dysfunction;

Known to suffer for mental illness;

Long-term use of sedatives and analgesics.

**Withdrawal**

Patients who wish to withdraw from the study can withdraw at any time. When the patients wish to withdraw, investigators may contact them by telephone or visiting for asking a reason for withdrawl, which should be recorded in the clinical research form.

**Randomization and concellation**

The study is performed as a double-blind randomized study. A computer-generated list of numbers will be used for drug allocation, and the list will be concealed in opaque sealed envelopes that will be numbered and opened sequentially. A nurse who will not be involved in any other part of the study will obtaine the envelopes and then prepare the premedications by drawing 1 μg∙kg^–1^ of dexemedetomidine into a 1-mL syringe and diluting it to a volume of 1 mL with 0.9% saline or the same volume of saline as a control.

**Blinding**

Patients, grouping designer, anesthesiologists, and assessors will be blinded to the study. A member of the research team will be responsible for recruitment of patients and their written informed consent. Once unblinding in exceptional circumstances, it will be recorded and analyzed in detail when study completed.

**Calculation of the number of patients**

Power analysis indicated that a sample size of 40 patients per group would be required to detect a 20% difference in time to extubation between control and treatment groups with a 5% type I error and a power of 0.9. Therefore, we decided to recruite 81 patients for the study.

**Methods**

81 patients will be randomly allocated into two groups, 41 patients in the placebo group and 40 patients in the dexmedetomidine group. At about 45 to 60 minutes before anesthesia induction, Patients will receive intranasal 1 μg∙kg^–1^ of dexmedetomidine or intranasal 1 mL with 0.9% saline. Test premedications were dripped into both nostrils of the patients in the supine position. Automatic sphygmomanometer will be used to measure blood pressure. Oxygen saturation and heart rate will be measured by a pulse oximeter. Respiratory rate, sedation scores and anxiety will be assessed regularly. Patients will be managed with protocol standardized general anesthesia with target-controlled infusion (TCI) of propofol and remifentanil, suspension laryngoscopy surgery and postoperative care.

**Endpoints**

**Primary end points**

Extubation time after intranasal dexmedetomidine premedications

**Secondary end points**

Sedation level: a 7-point modified Observer’s Assessment of Alertness/Sedation scale (OAA/S).

Anxiety levels using a 4-point anxiety score (1 = combative, 2 = anxious, 3 = calm, and 4 = amiable) rated by patients.

The occurrence of peri-operative adverse events: 1) hypertension (SBP increased 130% of the pre-operative value for more than 1 min), 2) hypotension (SBP decreased more than 30% of the pre-operative value for more than 1 min), 3)tachycardia (HR >100 bpm for more than 10 s), 4)bradycardia (HR <45 bpm for more than 10 s), 5)shivering, 6)nausea and vomiting, 7) for pain and analgesic demand within 2 h after extubation, 8) intraoperative awareness.

The target plasma concentrations of propofol and remifentanil at induction, before insertion, and upon removal of the operative laryngoscope at the return of spontaneous breathing, at emergence, and at extubation.

Recovery time: the time elapsed between the cessation of anesthetic infusions and the return of spontaneous breathing, emergence, and extubation.

Patient satisfaction using a 3-point satisfaction score (1 = highly satisfactory, 2 = acceptable, and 3 = unacceptable) rated by patients when discharged from the PACU.

**Study schedule**

Study start date: March 2014

Estimated study completion date: July 2014

Estimated primary completion date: June 2014

**Location**

Department of Anaesthesia, First Municipal People’s Hospital of Guangzhou, an Affiliate Hospital of Guangzhou Medical College, Guangzhou, China.

**Ethics and regulation**

This study protocol conformed to the ethical guidelines of the 1975 Helsinki Declaration and International Conference on Harmonisation of Technical Requirements of Pharmaceuticals for Human Use (ICH) Note for Guidance on Good Clinical Practice (ICH, Topic E6, 1995)

This study was approved by the Institutional Review Board of Severance Hospital.

Compensation: Intrnasal drugs and addministration will be free for participate patients in this study.

**References**

[1] NISHIYAMAT, MATSUKAWAT, HANAOKAK, et a1．The effects Of midazoiam age and gender on the optimal premedication dose of intramuscular midazoiam[J].Anesth Analg, 1998, 86

(5) 1103 -1108.

[2] Kain ZN et al.Anesth Analg,1997;84:427

[3] Haas C et al. Anaesthesist,1998;47:838

[4]段开明, 欧阳文, 王明安等. 咪唑安定对患者麻醉手术前身心应激的调控[J].临床麻醉学杂志, 2004, 20(4) ：219 -220.

[5]Bauer TM，Ritz R，Haberthur C，et a1．Prolonged sedation due to accumulation of conjugated metabolites of midazolam．Lancet，1995，346(8969)：145-147．

[6]孙勤，徐伟华，方强．咪达I唑仑住ICU机械通气患者的应用研究．浙江创伤外科，2006，11（2)：163—164．

[7]Massad IM, Mohsen WA, Basha AS, et al. A banlanced anesthesia with dexmedetomidine decreases postoperative nausea and vomiting after laparoscopic surgery[J]. Saudi Med J, 2009; 30(12):1537-41.

[8]E.G.Elvan,et al. Dexmedetomidine and postoperative shivering in patients undergoing elective abdominal hysterectomy[J]. European Journal of Anaesthesiology 2008;25:357-364

[9]Sato M, Shirakami G, Tazuke-Nishimura M, et al. Effect of single-dose dexmedetomidine on emergence agitation and recovery profiles after sevoflurane anesthesia in pediatric ambulatory surgery[J]. J Anesth 2010; 24:675– 682.

[10]Ozcengiz D, Gunes Y, Ozmete O. Oral melatonin, dexmedetomidine, and midazolam for prevention of postoperative agitation in children[J]. J Anesth 2011; 25:184–188.

[11]Bloor BC, Ward DS, Belleville JP, Maze M. Effects of intravenous dexmedetomidine in humans. II. Hemodynamic changes. Anesthesiology 1992;77:1134 – 42

[12] Maze M, Tranquilli W. Alpha-2 adrenoceptor agonists: defining the role in clinical anesthesia[J]. Anesthesiology 1991;74581-605

[13]Yuen VM, Hui TW, Yuen MK, Irwin MG. A double blind crossover assessment of the sedative and analgesic effects of intranasal dexmedetomidine. Anesth Analg 2007;105:374 – 80

[14]N. Nooh, S. A. Sheta, W. A. Abdullah, et al. Intranasal atomized dexmedetomidine for sedation during third molar extraction. Int. J. Oral Maxillofac.Surg. 2013; YIJOM-2611

[15]V. M. Yuen,T. W. Hui,M. G. Irwin, et al. A randomised comparison of two intranasal dexmedetomidine doses for premedication in children. Anaesthesia 2012, 67, 1210–1216

[16]Walker KJ, Smith AF. Premedication for anxiety in adult day surgery. Cochrane Database Syst Rev 2009: CD002192.

[17]Caumo W, Hidalgo MP, Schmidt AP, Iwamoto CW, Adamatti LC, Bergmann J, Ferreira MB. Effect of pre-operative anxiolysis on postoperative pain response in patients undergoing total abdominal hysterectomy. Anaesthesia 2002; 57: 740-6.
