# Supplementary material for: Intranasal Dexmedetomidine as a Sedative Premedication for Patients Undergoing Suspension Laryngoscopy: A Randomized Double-Blind Study
Source: PLoS One. 2016 May 19;11(5):e0154192. doi: 10.1371/journal.pone.0154192 (PMC4873234; doi:10.1371/journal.pone.0154192)
Supplement: S3 File — This is original protocol in Chinese (DOCX) [file pone.0154192.s004.docx]

**Protocol**

**支撑喉镜手术患者术前经鼻滴注右旋美托咪定的随机对照研究**

**研究背景：**

术前大部分患者存在不同程度的紧张、焦虑，这使患者的应激反应增强，影响麻醉和手术的正常进行，而且体内交感系统过度兴奋易致心血管不良事件，影响术后恢复。因此，术前使用一些镇静抗焦虑药物是非常必要的。术前用药是麻醉的重要组成部分，是麻醉的开始，良好的术前用药应具有镇静、抗焦虑、不良反应少等优点^[1]^ 。苯巴比妥钠作为传统术前用药,它有镇静不足、抗焦虑弱等缺点。术前用药，欧美较多国家(美国占75%，在德国占96%)^[2~3]^首选咪达唑仑，它在镇静、催眠、顺行性遗忘等方面都优于苯巴比妥钠^[4]^。但是，咪达唑仑伴有抑制呼吸、镇静过度、易诱发谵妄、使恢复期苏醒时间延长等缺点^[5~6]^。术前用药在无监测条件下存在一定的安全隐患，并且它可能导致苏醒延迟，从而局限了它的应用。

右旋美托咪定（Dexmedetomidine，Dex）是一种高选择性的α_2_-肾上腺素能受体激动剂，它具有镇静、抗焦虑、抑制交感神经兴奋、镇痛、节省麻醉药物和稳定血流动力学的作用，临床剂量对呼吸无抑制作用。在麻醉恢复期，Dex能减少恶心呕吐^[7]^、寒颤^[8]^、术后躁动的发生率^[9~10]^、减少对镇痛药物的需求，能提高麻醉恢复质量，改善患者对麻醉的满意度。因此，Dex的镇静、抗焦虑、呼吸无抑制等优点使其作为术前用药有一定优势。

但是，Dex对循环系统的影响局限了其应用。应用Dex后，心率呈剂量依赖性下降，血压呈剂量依赖性的双相变化。Dex在作为术前用药有多种给药方式，如静脉注射、肌肉注射、经鼻滴注等。静脉注射Dex对循环系统的影响较大，且持续输注可能影响全麻苏醒时间。研究表明，静注0.25~2 μg∙kg^–1^（给药时间超过2分钟）引起剂量依赖性的血压下降14%~27%^[11]^。Lawrence等单次静脉预注2.0μg∙kg^–1^ Dex发现输注的第一分钟和第五分钟心动过缓的发生率明显增高（HR40﹤bpm）^[12]^。而且，静脉注射与肌肉注射给药方式是有创的，与目前提倡无创术前给药方法相悖。Dex无色无味，无黏膜刺激作用，经鼻滴注给药方便，患者接受度、舒适度好。Yuen等研究表明：75%、92%的健康成年志愿者在经鼻滴注1μg∙kg^–1^、1.5μg∙kg^–1^Dex后产生有意义的镇静作用^[13]^。N. Nooh 等研究表明经鼻滴注1.5μg∙kg^–1^的Dex作为第三磨牙拔除的镇静药物是有效、方便和安全的^[14]^。还有研究表明小儿经鼻滴注1μg∙kg^–1^、2μg∙kg^–1^ Dex分别产生53%、66%的满意镇静^[15]^。

对于成人，使用术前镇静药可能会有延迟苏醒的顾虑。但是，Cochrane等系统评价了17个比较术前用药抗焦虑药（苯二氮类，阿片类药物和β受体阻滞剂）与安慰剂的随机对照临床试验，结果显示没有证据证明术前抗焦虑药物的使用会延迟日间手术的出院时间^[^[^17^](#_ENREF_17)^]^。恰当的术前用药能节省麻醉药和镇痛药的使用，从而减少了它们的副作用如术后恶性呕吐等，最终使择期手术的患者受益颇多^[^[^18^](#_ENREF_18)^]^。Dex是否会延迟苏醒及它们之间的关系如何是未知的，目前并没有研究探究Dex作为术前镇静抗焦虑药与苏醒延迟之间的联系。

因此，本研究的目的在于研究术前经鼻滴注右美托咪定的镇静效果、安全性及对全麻苏醒期的影响。

**研究设计：**

本研究为单中心、前瞻性、随机、双盲、对照设计，实验方案将申请广州医科大学附属广州市第一人民医院伦理委员会审查批准，并将在clinical trail网站注册。符合入组条件患者术前随机经鼻滴注右旋美托咪定或安慰剂，并签署相关知情同意书。

研究类型： 介入。

分配：随机。

终结点分类：安全与效果研究。

干预模式：平行分组。

盲法：双盲 （患者和评估者）。

**资格**

年龄资格研究：18–60岁

性别资格： 两个

接受健康志愿者： 无

**标准**

纳入标准：择期行支撑喉镜声带息肉摘除术患者

体重指数（BMI）<30kg/m^2^

年龄18–60岁

ASA I-II级

排除标准：拒绝参加该研究者；

对右旋美托咪定或其他麻醉药过敏者；

既往患有心脏病史；

妊娠的女性；

无可靠避孕措施的绝经妇女;

术前心率小于45bpm；

Ⅱ或Ⅲ度房室传导阻滞；

缺血性心脏病；

正在服用降压药如甲基多巴、可乐定或其它α_2_受体激动剂；

哮喘患者；

睡眠呼吸暂停综合征患者；

肝肾功能障碍患者；

已知患有精神疾病者；

长期服用镇静药及镇痛药患者.

**患者中途终止试验**

患者具有随时退出试验的权利。患者中途终止试验的原因应以电话或随访的形式记录下来。

**随机**

将由研究小组的一位成员负责执行随机。根据电脑产生随机序列分为两组，装入连续编号的不透明信封并密封。分组遮蔽直到统计分析完成。大约在麻醉前45~60min，每个患者随机经鼻滴注右旋美托咪定（1μg.kg^-1^）或0.9%生理盐水安慰剂。使用1毫升注射器将右旋美托咪定(100μg.ml^-1^静脉剂型)以0.9%生理盐水稀释为1 毫升终容量。患者于仰卧位从两个鼻孔滴入1 毫升研究药物或安慰剂。一位专门护士负责保存不透明的信封和准备研究药物，但不参与任何其它试验内容。

**盲法**

根据顺序编号募集患者，患者、分组设计者和评估者均对分组持盲。将由一位研究小组的成员负责募集患者，并签署患者书面麻醉知情同意书和受试者知情同意书。一旦在特殊情况下破盲，将会详细记录和分析。

**样本大小估算**

样本量大小根据统计功效分析，为了使试验组与对照组的拔管时间有20%的差异，以及有90%机会检验出且I类错误发生率为5%。假设缺失率为5%，则最终的样本量为80个，本研究选取81例患者纳入研究。

**方法**

81例患者随机分为两组，经鼻滴注右旋美托咪定组40例，对照组41例。麻醉诱导前45~60分钟，患者随机经鼻滴注右旋美托咪定（1μg.kg^-1^）或0.9%生理盐水安慰剂。使用1毫升注射器将右旋美托咪定(100μg.ml^-1^静脉剂型)以0.9%生理盐水稀释为1 毫升终容量。患者于仰卧位从两个鼻孔滴入1毫升研究药物或安慰剂。血压是自动血压计测量，血氧饱和度及心率由脉搏氧饱和度仪测定。呼吸频率、镇静评分和焦虑水平定时评定。

患者的标准TCI输注的异丙酚和瑞芬太尼全身麻醉方式、支撑喉镜声带息肉摘除术和术后护理、标准监测均统一。

**研究终点**

**主要终点：**

主要终点：苏醒拔管时间。

**次要终点：**

镇静水平：使用7分制改良观察者评估的警觉/镇静评分（OAA/S）；

焦虑水平：4分制患者焦虑评分（1分恐惧，2分焦虑，3分安静，4分舒适）；

围术期不良事件的发生情况包括：1）低血压（收缩压降低基础水平30%以上达1分钟），2）高血压（收缩压升高基础水平30%以上达1分钟），3）心动过缓（心率低于45次/分达10秒以上），4）心动过速（心率高于100次/分达10秒以上），5）术后寒战的发生，6）术后恶心、呕吐的发生，7）术后2小时内出现疼痛（疼痛视觉评分大于50）及对镇痛药的需求，8）术中知晓；

术中异丙酚和瑞芬太尼TCI血浆药物浓度；

术后自主呼吸恢复、睁眼和气管拔管时间；

患者对麻醉和手术的总体满意度（1分非常满意，2分可以接受，3分不满意）。

**统计学处理**

数据表示为平均值与标准差（M±SD），用双样本t检验进行统计分析。非参数数据如镇静评分、焦虑评分和患者满意度表示为中位数与四分位范围，用曼-惠特尼检验进行统计分析。小样本数据的发生率比较应用χ^2^和Fisher精确检验统计分析。P< 0.05认为具有统计学差异。

**研究计划**

开始研究时间：2014年3月

预计研究完成时间：2014年7月

预计初步完成研究时间：2014年6月

**研究地点**

广州医科大学附属市第一人民医院

**伦理**

本项目方案谨遵赫尔辛基宣言和人类用药注册技术要求国际协调会议相关指引，经由所在单位伦理委员会审查和批准。

滴鼻途径给药和药液均对患者免费。

**参考文献：**

[1] NISHIYAMAT, MATSUKAWAT, HANAOKAK, et a1．The effects Of midazoiam age and gender on the optimal premedication dose of intramuscular midazoiam[J].Anesth Analg, 1998, 86

(5) 1103 -1108.

[2] Kain ZN et al.Anesth Analg,1997;84:427

[3] Haas C et al. Anaesthesist,1998;47:838

[4]段开明, 欧阳文, 王明安等. 咪唑安定对患者麻醉手术前身心应激的调控[J].临床麻醉学杂志, 2004, 20(4) ：219 -220.

[5]Bauer TM，Ritz R，Haberthur C，et a1．Prolonged sedation due to accumulation of conjugated metabolites of midazolam．Lancet，1995，346(8969)：145-147．

[6]孙勤，徐伟华，方强．咪达I唑仑住ICU机械通气患者的应用研究．浙江创伤外科，2006，11（2)：163—164．

[7]Massad IM, Mohsen WA, Basha AS, et al. A banlanced anesthesia with dexmedetomidine decreases postoperative nausea and vomiting after laparoscopic surgery[J]. Saudi Med J, 2009; 30(12):1537-41.

[8]E.G.Elvan,et al. Dexmedetomidine and postoperative shivering in patients undergoing elective abdominal hysterectomy[J]. European Journal of Anaesthesiology 2008;25:357-364

[9]Sato M, Shirakami G, Tazuke-Nishimura M, et al. Effect of single-dose dexmedetomidine on emergence agitation and recovery profiles after sevoflurane anesthesia in pediatric ambulatory surgery[J]. J Anesth 2010; 24:675– 682.

[10]Ozcengiz D, Gunes Y, Ozmete O. Oral melatonin, dexmedetomidine, and midazolam for prevention of postoperative agitation in children[J]. J Anesth 2011; 25:184–188.

[11]Bloor BC, Ward DS, Belleville JP, Maze M. Effects of intravenous dexmedetomidine in humans. II. Hemodynamic changes. Anesthesiology 1992;77:1134 – 42

[12] Maze M, Tranquilli W. Alpha-2 adrenoceptor agonists: defining the role in clinical anesthesia[J]. Anesthesiology 1991;74581-605

[13]Yuen VM, Hui TW, Yuen MK, Irwin MG. A double blind crossover assessment of the sedative and analgesic effects of intranasal dexmedetomidine. Anesth Analg 2007;105:374 – 80

[14]N. Nooh, S. A. Sheta, W. A. Abdullah, et al. Intranasal atomized dexmedetomidine for sedation during third molar extraction. Int. J. Oral Maxillofac.Surg. 2013; YIJOM-2611

[15]V. M. Yuen,T. W. Hui,M. G. Irwin, et al. A randomised comparison of two intranasal dexmedetomidine doses for premedication in children. Anaesthesia 2012, 67, 1210–1216

[16]Walker KJ, Smith AF. Premedication for anxiety in adult day surgery. Cochrane Database Syst Rev 2009: CD002192.

[17]Caumo W, Hidalgo MP, Schmidt AP, Iwamoto CW, Adamatti LC, Bergmann J, Ferreira MB. Effect of pre-operative anxiolysis on postoperative pain response in patients undergoing total abdominal hysterectomy. Anaesthesia 2002; 57: 740-6.
