# Supplementary material for: Intranasal Dexmedetomidine as a Sedative Premedication for Patients Undergoing Suspension Laryngoscopy: A Randomized Double-Blind Study
Source: PLoS One. 2016 May 19;11(5):e0154192. doi: 10.1371/journal.pone.0154192 (PMC4873234; doi:10.1371/journal.pone.0154192)
Supplement: S4 File — This is original Informed Consent Form for this trial. (DOCX) [file pone.0154192.s005.docx]

**Informed Consent Form**

**受试者知情同意书**

尊敬的女士/先生：

我们诚挚邀请您参加“支撑喉镜手术患者术前经鼻滴注右旋美托咪定的随机对照研究”，这是一项高质量的前瞻、随机、对照临床研究观察项目，本项目已经过本单位伦理委员会审查批准，我们保证您参加本临床研究的相关安全将受到充分保障。

1.研究目的：

支撑喉镜手术患者术前经鼻滴注右美托咪定的镇静和麻醉辅助效应。

2. 研究手段：

术前大部分患者存在不同程度的紧张、焦虑，易致心血管不良事件，影响术后恢复。因此，术前使用一些镇静抗焦虑药物是非常必要的。苯巴比妥钠作、咪达唑仑等作为术前用药，在无监测条件下存在一定的安全隐患，并且可能导致苏醒延迟，从而局限了其应用。右旋美托咪定是一种高选择性的α_2_-肾上腺素能受体激动剂，它具有镇静、抗焦虑、抑制交感神经兴奋等作用，临床剂量对呼吸无抑制作用。这些优点使其作为术前用药有一定优势。右旋美托咪定无色无味，无黏膜刺激作用，经鼻滴注给药方便，患者接受度、舒适度好。小儿人群数据显示，经鼻滴注右旋美托咪定能产生良好的镇静作用，且因其血浆浓度逐渐缓慢的增加，有可能减弱其对血压、心率的影响，从而能保证术前用药的安全性。本项目拟对行支撑喉镜声带息肉摘除术的患者术前经鼻滴注右旋美托咪定，观察其作为麻醉术前用药的有益效果。

本项目直接相关的仪器、设备、耗材和人工等费用共约300￥/人，将从项目组负责人阮祥才主任的科研经费支出，不会增加您的费用。

3.参加研究的适宜人群：

拟行支撑喉镜声带息肉摘除术的患者，男女不限。

入选标准：年龄为18–60岁，美国麻醉医师协会（ASA）分级I-II级。

排除标准：拒绝参加该研究者；对麻醉药过敏者；既往患有心脏病史；妊娠女性；无可靠避孕措施妇女；病态肥胖症（体重指数≥35kg.cm^-2^）；术前心率小于45bpm；Ⅱ或Ⅲ度房室传导阻滞；缺血性心脏病；正在服用降压药如甲基多巴、可乐定或其它α2受体激动剂；哮喘患者；睡眠呼吸暂停综合征患者；肝肾功能障碍患者；已知患有精神疾病者；长期服用镇静药及镇痛药患者；

募集步骤：符合标准病患如意愿参与本实验，将首先签署本研究相关知情同意文书；其次详细登记患者姓名、年龄、职业、联系方式、发病时间、既往就诊史、合并症及其治疗情况等。

4.试验方法：

符合纳入标准的患者根据纳入序号随机进入①右旋美托咪定或②安慰剂组。患者于麻醉前45~60分钟经鼻滴注无色、无味且无黏膜刺激的右旋美托咪定（1 μg.kg^-1^）或0.9%生理盐水安慰剂。所有患者按照常规行全身麻醉、支撑喉镜声带息肉摘除术和术后护理。所有患者均接受标准的监测。

经鼻给药后麻醉医生会对您的生命体征及镇静焦虑水平进行定时评分，术后将对您的不良事件的发生及认知功能进行评估，术后24小时将随访。整个评估和随访过程都是无创的，也不需抽取任何血液或其他组织标本，无需做额外的实验室检查，因此不会额外增加您的费用。而且，根据现有的研究资料，术后早期不良事件的处理、认知功能评估和术后麻醉随访，可以更好地促进麻醉和手术后康复。

5.参加本次实验研究的受益和风险：

（1）受试者收益：

1）免费的干预药物和监测；2）减少术中麻醉用药；3）减少术后躁动发生率，提高患者舒适性；4）减少术后镇痛阿片类药的使用；5）减少术后恶心、呕吐的发生率；6）术后早期认知功能评估和术后麻醉随访，可以更好地促进麻醉和手术后康复。常规的麻醉药物、监测和治疗费用仍会出现在您的账单中。

（2）可能的风险

您有50%的机会可能被分配入对照组，接受目前本专业经典的治疗策略所指导的全身麻醉方案，实施方法完全符合临床常规。本研究设计完全符合临床常规，不会增加您的医疗风险。一旦出现相关不良事件，我们保证通过监测早发现、早治疗和全称跟踪，并在解决相关不良事件之后，与患方协商解决费用与经济补偿，该类费用将由项目负责人阮祥才主任的科研经费支付，不会增加您的经济负担。

6.补偿机制：

我们会尽力保证您在研究阶段的安全。但如果项目的实施造成对您不利的不良事件，我们会积极治疗和补救。您的相关药物和治疗费用将由我们从科研经费中支付，至于极低的几率下，对您精神和心理造成不良影响，我们也会通过跟您协商的方式达成一个合理的补偿措施。

7.研究记录的保密:

我们对您的身份的记录和个人资料完全保密，有关识别您的医疗记录也会保密，您的姓名和个人信息不会出现在研究报告和出版物中。

8.受试者的权利:

参加本研究是自愿的，您可以拒绝参加，或在研究的任何阶段随时退出，您的医疗待遇和权益不受任何影响。

9.受试者声明：

我已详细阅读了本知情同意术提示的研究内容，我的医生已向我作了详尽的说明，我完全了解参加本研究的目的，性质，方法及我的权益与风险。得知我的个人资料是受到保密的，隐私权也得到保护。我愿意参加本研究，并同意按照研究方案和知情同意书提示配合医生。

受试者签名： 联系电话： 日期： 年 月 日

受试者监护人签名（备选）： 联系电话： 日期： 年 月 日

我确认已向受试者解释了本研究的详细情况，包括可能的获益和风险。

医生签名： 联系电话： 日期： 年 月 日
